# Pulmonary actinomycosis as a cause of chronic productive cough in a heavy smoker male with poor dental hygiene: A case report

**DOI:** 10.1002/ccr3.6031

**Published:** 2022-07-11

**Authors:** Musaed Alsamawi, Abdulrahman F. Al‐Mashdali, Yasser Eldeeb

**Affiliations:** ^1^ Department of Infectious Diseases Hamad Medical Corporation Doha Qatar; ^2^ Department of Internal Medicine Hamad Medical Corporation Doha Qatar

**Keywords:** antibiotics, case report, computed tomography, endobronchial biopsy, pulmonary actinomycosis

## Abstract

Pulmonary actinomycosis is a rare chronic granulomatous bacterial disease caused by Actinomyces species. Given its nonspecific clinical and radiological manifestations, the diagnosis might be delayed or even missed. Pulmonary actinomycosis mimics tuberculosis, aspergillosis, or malignancy both clinically and radiographically, and it should be considered in patients with chronic lung diseases.

## Graphical abstract

Pulmonary actinomycosis should be considered in the differential diagnoses of chronic infectious lung disease. Chronic productive cough with lung infiltrates on imaging might be the only manifestation of this condition.

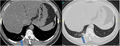

## 1 INTRODUCTION

Actinomycosis is a rare, chronic granulomatous disease caused by anaerobic gram‐positive bacteria, Actinomyces species, that typically colonize the mouth, colon, and genital tract.^1^ Human is the natural reservoir, and there is no person‐to‐person transmission of the disease.^1^ Given their low virulence, Actinomyces require disruption of mucosa to cause the disease.^2^ Cervicofacial and abdominopelvic actinomycosis is the most common site of actinomycosis.^2^ The pulmonary form of actinomycosis is the third most common type of actinomycosis (approximately 15% of the cases). It usually affects males in their fourth and fifth decades of life.^3^ The clinical presentation of pulmonary actinomycosis is highly nonspecific, mimicking other infectious diseases, such as bacterial pneumonia, tuberculosis, and aspergillosis.^1^, ^3^ Also, actinomycosis is characterized by its tendency to mimic malignant conditions due to its ability to invade surrounding tissues and form masses. Accordingly, the diagnosis of actinomycosis is usually delayed or even missed.^1^, ^2^, ^3^ Herein, we describe an unusual case of pulmonary actinomycosis that was initially misdiagnosed as a bacterial infection in multiple hospital visits.

## 2 CASE PRESENTATION

A 42‐year‐old man with no significant past medical history presented to our emergency department (ED) with a six‐month history of productive cough. He sought medical advice a few months before this presentation because of a similar complaint. At that time, he was diagnosed with boronchial asthma due to the presence of wheezing on chest examination. He was given salbutamol and oral antibiotic and discharged from the ED. Again, he presented with a productive cough increasing despite using salbutamol. Inhaled steroids and moxifloxacin were prescribed, and he was discharged. He reported a daily cough of yellowish sputum at the current presentation, which sometimes mixed with blood. He denied any chest pain, shortness of breath, weight loss, night sweating, or loss of appetite. He was a heavy cigarette smoker (3–4 packs/day) for 30 years. He denied alcohol intake, personal, or family history of lung diseases.

**FIGURE 1:**
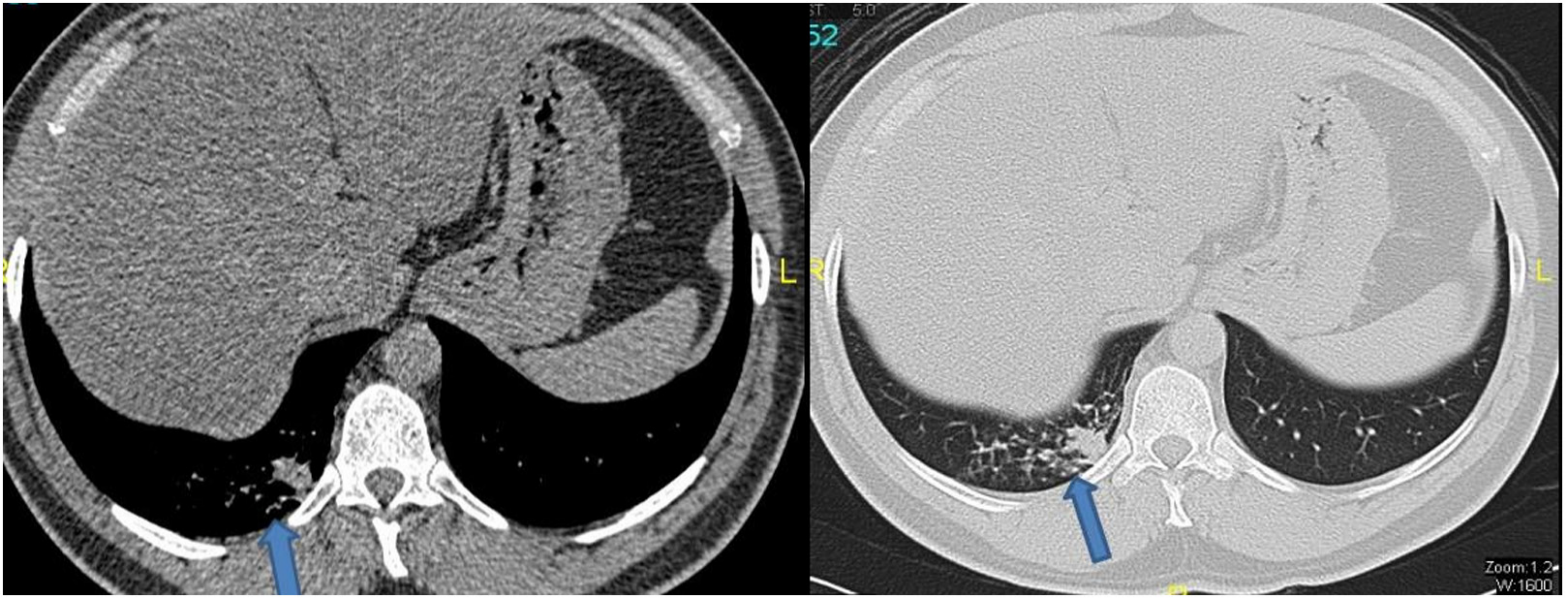
CT of the chest showing peripheral soft tissue density along the posterior basal segment of the right lung

On physical examination, he was afebrile, not tachypneic or tachycardic, and breathing room air. Oral cavity examination revealed poor oral hygiene, due to heavy smoking, with most of his teeth extracted. Chest examination revealed coarse crackles in the right lower chest zone. There was no lymphadenopathy or organomegaly. Other systemic examination was unremarkable. His laboratory results were significant for hemoglobin of 14.7 g/dl, white blood cells (WBC) of 9300/μl, creatinine of 81 mmol/L, alanine transaminase (ALT) of 62 U/L, and aspartate transaminase (AST) of 26 U/L. Computed tomography (CT) of the chest depicted a peripheral soft tissue density along the right posterior basal segment of the lung, suggestive of a granulomatous or neoplastic process (Figure 1). Accordingly, he underwent bronchoscopy with endobronchial wash and biopsy from the right lower lobe of the lung.

**FIGURE 2:**
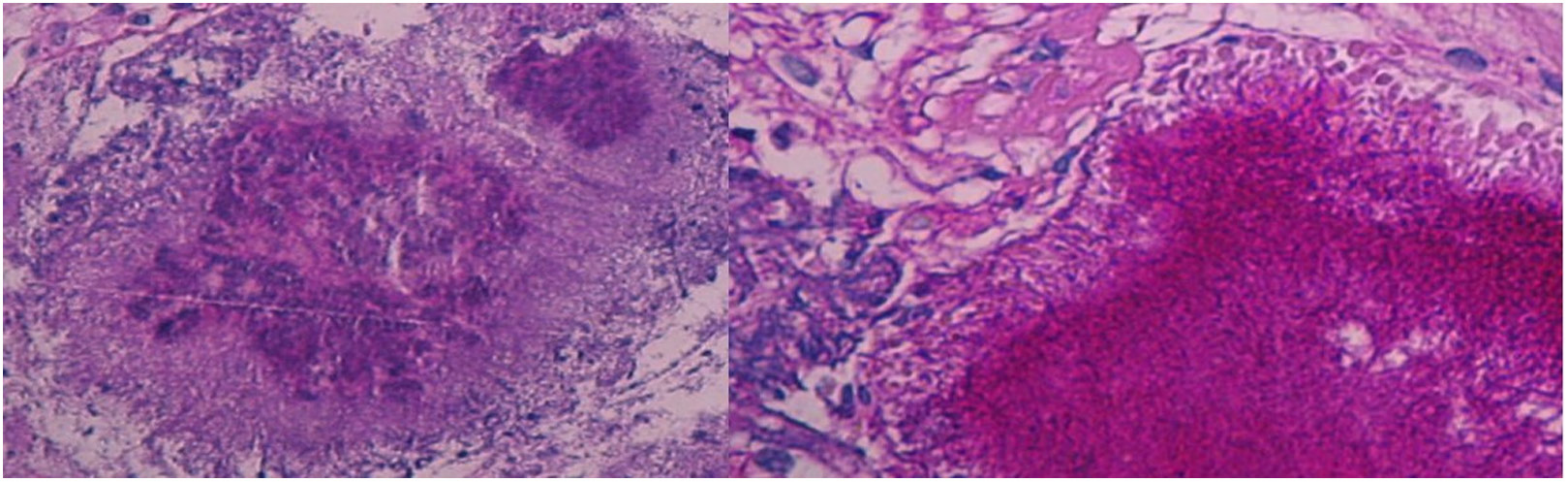
Histopathological examination of the lung tissue (endobronchial tissue biopsy) is consistent with pulmonary actinomycosis

Cytology (from the bronchial wash) was negative for malignant cells. However, histopathological examination showed inflammatory exudate and colonies of Actinomyces (Figure 2). Unfortunately, the microbiological culture was not done because the pulmonologist/bronchoscopist was thinking about malignancy, given the patient history of chronic cough and heavy smoking, so the tissue sample was not sent to the microbiology laboratory for further identification. Meanwhile, the acid‐fast bacillus (AFB) and fungal stains were negative. The biopsy findings showed no evidence of malignancy or granulomatous inflammatory disease. Of note, the human immunodeficiency virus (HIV) test was negative. Accordingly, a diagnosis of pulmonary actinomycosis was made. Treatment with intravenous ceftriaxone 2 g per day for the initial 6 weeks was applied, followed by amoxicillin administered orally in a dose of 500 mg three times a day for 18 weeks (total of 26 weeks). The patient improved significantly over that period, and his chronic productive cough nearly resolved. In addition, dental care was started with regular follow‐up with the dentist. A follow‐up CT chest was requested as an outpatient; however, the patient did not do it.

## 3 DISCUSSION

Actinomycosis is an infection caused by anaerobic gram‐positive bacteria from Actinomyces species. Actinomyces spp. is higher prokaryotic bacteria belonging to the family Actinomyceataceae. They were misclassified as fungi at the beginning of the 19th century.^4^ The word “actinomycosis” was derived from the Greek terms aktino, which describes the radiating appearance of the sulfur granule, and mykos, which means a mycotic disease.^2^ The most commonly isolated pathogen is *Actinomyces israelii*. In cases of pulmonary involvement, *Actinomyces meyeri* is also frequently found.^3^, ^5^, ^6^ These bacteria are typically found in the human oral cavity. The disease usually develops due to the aspiration of pathogens from oropharyngeal or gastrointestinal secretions.^1^, ^2^ Individuals with advanced dental caries are particularly predisposed, as in our patient. Development of the disease is also promoted by injury of the oral cavity and aspiration, including foreign body aspiration.^2^ The frequency of actinomycosis is higher among people with immunodeficiency, malnutrition, radiotherapy, diabetes mellitus, or liver diseases.^6^

The bacteriological identification of Actinomyces proves the diagnosis of actinomycosis. Characteristically, colonies of Actinomyces appear as “molar‐tooth” or “bread‐crumb” colonies in broth media after at least 5 days of incubation. Most of the literature classifies the tissue response as granulomatous or “granulomatoid‐like,” although giant cells and granulomata are rarely seen. Sulfur granules are a characteristic feature of the disease. These granules are round or oval basophilic masses with a radiating arrangement of eosinophilic clubs on the surface; they sometimes can be seen even with a magnifying glass.^2^, ^7^, ^8^ The name “sulfur granule” has its origin in the small nodules resembling elemental sulfur that were commonly used in pharmaceuticals in the 19th century.^9^ Recently, molecular methods, such as real‐time PCR fluorescence in situ hybridization (FISH), have been issued for the early identification of Actinomyces species.^10^

Pulmonary actinomycosis is a difficult condition to diagnose. Even among experienced physicians, it might be misdiagnosed as tuberculosis, lung abscess, or lung cancer.^11^, ^12^ Typical respiratory symptoms of patients with thoracic actinomycosis are cough (84%), sputum (74%), chest pain (68%), dyspnea (47%), hemoptysis (31%), and localized chest‐wall swelling (10%). Systemic symptoms are weight loss (53%) malaise (42%), night sweats (32%), and fever (21%). Marked weight loss, malaise, and high fever may suggest disseminated disease.^2^ Physical signs are nonspecific, except in advanced, untreated disease, when sinuses and fistulae may suggest the diagnosis. The findings are occasionally those of the associated complications, such as pleural effusion or empyema.^3^, ^6^, ^11^ The disease usually shows a peripheral and lower lobe predominance, probably reflecting the role of aspiration in its pathogenesis.^11^ The CT is probably more helpful than the plain radiograph, particularly if performed with a bone window display, which better delineates minimal bony change, such as early rib erosion and osteomyelitis. The radiological findings of pulmonary actinomycosis are nonspecific and highly variable, including patchy consolidation, multifocal nodular appearances, cavitary lesions, pleural thickening, pleural effusion, and hilar/mediastinal lymphadenopathy.^13^ Fiberoptic bronchoscopy is usually not diagnostic in pulmonary actinomycosis unless there is an evident endobronchial disease on which biopsy can be performed.^3^, ^11^

The optimal treatment for pulmonary actinomycosis is antibiotic therapy for a prolonged period. The recommended antibiotic is intravenous penicillin G for 2–6 weeks, followed by oral penicillin V for at least 6 months. However, other β‐lactams, such as amoxicillin and ceftriaxone, can be used for the treatment of actinomycosis.^1^, ^3^ Importantly, management of the existing predisposing factors, including poor dental hygiene, should not be ignored to prevent disease recurrence. The adverse effects of tobacco smoking on oral health should be explained to smoker patients, and smoking cessation should be highly encouraged. Smoking can lead to discoloration of teeth and dental restorations, bad breath, taste and smell disorders, impaired wound healing, and periodontal disease.^14^ Untreated pulmonary actinomycosis can lead to severe complications, including empyema necessitans, ribs destruction, and even the invasion of the adjacent heart.^11^ Generally, the prognosis of pulmonary actinomycosis is less favorable compared with the other commoner forms, such as cervicofacial and abdominal disease. This may be related to the greater incidence of disseminated disease in the thoracic form and may also reflect the delayed diagnosis in such a condition.^5^ However, when early and proper treatment is given, the condition has an excellent prognosis with a very low mortality rate.^1^, ^3^, ^9^

## 4 CONCLUSION

Actinomycosis, especially in pulmonary form, is a rare disease. However, it should remain an important differential during the investigation of chronic infiltrative lung diseases. The early diagnosis allows for proper medical treatment and saves patient from unnecessary surgery.

## AUTHOR CONTRIBUTIONS

MA collected the data, reviewed the literature, and wrote the manuscript. AA collected the data and wrote the manuscript. YE reviewed and edited the final manuscript. All authors reviewed and approved the final version of the article.

## CONFLICT OF INTEREST

The authors have no conflict of interest to declare.

## ETHICAL APPROVAL

This case report was approved by the Hamad Medical Corporation's Medical Research Center.

## CONSENT

Written informed consent was obtained from the patient for the publication of this case report.

## Data Availability

The datasets used and/or analyzed during the current study are available from the corresponding author on request.
